# Temporal trends in hypertension related end stage renal disease mortality rates: an analysis of gender, race/ethnicity, and geographic disparities in the United States

**DOI:** 10.3389/fneph.2023.1339312

**Published:** 2024-01-15

**Authors:** Adarsh Raja, Sandesh Raja, Shafin Bin Amin, Madiha Salman, Bazil Azeem, Laksh Kumar

**Affiliations:** ^1^ Department of Medicine, Shaheed Mohtarma Benazir Bhutto Medical College Lyari, Karachi, Pakistan; ^2^ Department of Medicine, Dow Medical College, Dow University of Health Sciences, Karachi, Pakistan

**Keywords:** hypertension, renal failure, mortality, renovascular hypertension, end-stage renal disease

## Abstract

**Background:**

According to one USA Renal Data System report, 57% of end-stage renal disease (ESRD) cases are attributed to hypertensive and diabetic nephropathy. Yet, trends in hypertension related ESRD mortality rates in adults ≥ 35 years of age have not been studied.

**Objectives:**

The aim of this retrospective study was to analyze the different trends hypertension related ESRD death rates among adults in the United States.

**Methods:**

Death records from the CDC (Centers for Disease Control and Prevention Wide-Ranging OnLine Data for Epidemiologic Research) database were analyzed from 1999 to 2020 for hypertension related ESRD mortality in adults ≥ 35 years of age. Age-Adjusted mortality rates (AAMRs) per 100,000 persons and annual percent change (APC) were calculated and stratified by year, sex, race/ethnicity, place of death, and geographic location.

**Results:**

Hypertension-related ESRD caused a total of 721,511 deaths among adults (aged ≥ 35 years) between 1999 and 2020. The overall AAMR for hypertension related ESRD deaths in adults was 9.70 in 1999 and increased all the way up to 43.7 in 2020 (APC: 9.02; 95% CI: 8.19-11.04). Men had consistently higher AAMRs than woman during the analyzed years from 1999 (AAMR men: 10.8 vs women: 9) to 2020 (AAMR men: 52.2 vs women: 37.2). Overall AAMRs were highest in Non-Hispanic (NH) Black or African American patients (45.7), followed by NH American Indian or Alaska Natives (24.7), Hispanic or Latinos (23.4), NH Asian or Pacific Islanders (19.3), and NH White patients (15.4). Region-wise analysis also showed significant variations in AAMRs (overall AAMR: West 21.2; South: 21; Midwest: 18.3; Northeast: 14.2). Metropolitan areas had slightly higher AAMRs (19.1) than nonmetropolitan areas (19). States with AAMRs in 90th percentile: District of Columbia, Oklahoma, Mississippi, Tennessee, Texas, and South Carolina, had roughly double rates compared to states in 10th percentile.

**Conclusions:**

Overall hypertension related ESRD AAMRs among adults were seen to increase in almost all stratified data. The groups associated with the highest death rates were NH Black or African Americans, men, and populations in the West and metropolitan areas of the United States. Strategies and policies targeting these at-risk groups are required to control the rising hypertension related ESRD mortality.

## Introduction

Hypertension, often regarded as a silent killer, plays a pivotal role in the intricate web of factors leading to End-Stage Renal Disease (ESRD) (1). The kidneys, delicate filtration powerhouses, face relentless pressure when hypertension is unbridled. Over time, persistent high blood pressure damages the small blood vessels in the kidneys, impairing their ability to effectively filter waste and excess fluids (2). As the renal function declines, the risk of developing ESRD escalates. This intricate dance between hypertension and renal deterioration underscores the critical importance of blood pressure management in preventing the progression to ESRD (3). Hypertension related ESRD is a condition that occurs as a renal disease with hypertension (1). It is secondary to long-standing uncontrolled hypertension; data suggest it is a leading cause of end-stage renal disease or diabetic kidney disease (2). Hypertension related ESRD can be found with or without chronic kidney disease (CKD) with varying prevalence. According to this study (3), hypertension occurs in 23.3% of individuals without CKD, 35.8% of stage 1, 48.1% of stage 2, 59.9% of stage 3, and 84.1% of stage 4-5 CKD patients. Drug-induced hypertension can also cause hypertension related ESRD; therefore, the agents that cause hypertension can be significant; agents that can vary from NaCl to different drugs can cause hypertension (4). The use of cocaine, which can elevate blood pressure levels, can also cause hypertension related ESRD, according to this study (5). Rhabdomyolysis and vasoconstriction are well-known methods of pathology to cause acute renal failure and associated hypertension (6). According to one USA Renal Data System report, 57% of end-stage renal disease (ESRD) cases are attributed to hypertensive and diabetic nephropathy (7), highlighting a connection between hypertension and ESRD. Also, heart failure and renal dysfunction are common in the Western Hemisphere and the USA (8). Hypertensive ESRD accounts for 25% of new ESRD cases yearly in the USA, with more patients from Black Americans (9); also, the prevalence of ESRD is 3.5 times greater in Asian Americans and Alaska Natives (10). From 1991 to 2000, in the USA, the population of ESRD increased by around 186,000 new cases (11). In hypertension-related chronic kidney disease, there’s also a rise of 161.97% from 1990 to 2019, and the trend is likely to go upward for the next 25 years (12). Given these different factors that can be associated with Hypertension related ESRD and agents or substances that can cause it and its associated conditions, it is necessary to find a pattern in causation and associated conditions. Therefore, both demographic trends and mortality trends are examined.

## Methods

### Population and study setting

The comprehensive investigation relies on the use of the Centres for Disease Control and Prevention Wide-Ranging OnLine Data for Epidemiologic Research (CDC WONDER) database to collect information from death certificates. The study looked at instances of persons who died from Hypertension related ESRD between 1999 and 2020. The study used the diagnostic codes I12.0, I12.9, I13.0, I13.1, I13.2, and I15.0 from the 10th version of the International Classification of Diseases and Related Health Problems (ICD-10). The collection comprises information from death certificates from all 50 states and the District of Columbia regarding the causes of death. This tool has previously proven valuable in numerous studies investigating trends in mortality related to cardiovascular issues. The study looked at death records from the Multiple Causes of Death Public Use registry to find cases of hypertension related ESRD. Hypertension related ESRD was either a contributing factor or the major cause of death in these situations. In this study, Adults were categorized as people aged 35 and higher, ranging from 35 to 85 years old and above at the time of their demise. Comparable age criteria have been employed in previous research to classify individuals as adults (7, 13–15). The research did not necessitate approval from a regional institutional review board because it relied on deidentified public use data issued by the government. The STROBE standards for reporting observational research were followed in this study.

### Data abstraction

The dataset encompasses population counts, year of occurrence, location of death, demographic characteristics, geographical segmentation, state-specific data, as well as differentiation between urban and rural areas. Individuals die away in a variety of locations, including hospitals, households, hospices, nursing homes, and long-term care facilities. The phrase “demographics” pertains to data concerning gender, age, race, and ethnicity. The categories for race and ethnicity in this context include White non-Hispanics, Black or African Americans, Latino non-Hispanics, American Indians or Alaska Natives non-Hispanics, and Asian non-Hispanics or Pacific Islanders non-Hispanics. The data utilized in the analysis is derived from death certificates, which also served as a source in previous research that used the WONDER database (16). In accordance with the National Center for Health Statistics Urban-Rural Classification Scheme, the population was categorized into urban areas, which encompassed large metropolitan areas with a population of 1 million or more, as well as medium/small metropolitan areas with a population ranging from 50,000 to 999,999. Furthermore, rural regions were classified as those with fewer than 50,000 people, and there were additional counties as well, as defined by the 2013 U.S. Census (17). Based on the criteria set forth by the United States Census Bureau, the Northeast, Midwest, South, and West areas were divided into four main geographical categories (18).

### Statistical analysis

We computed mortality rates per 100,000 individuals for both unadjusted and age-adjusted data across the period from 1999 to 2020 to investigate regional trends in mortality related to hypertension related ESRD. These rates were divided into categories based on year, gender, race/ethnicity, state, and urban/rural status, and the 95% confidence intervals (CIs) were shown alongside them. The crude mortality rates were determined by dividing the total number of hypertension related ESRD deaths by the corresponding U.S. population for each respective year. Age-adjusted mortality rates (AAMR) were calculated by standardizing hypertension related ESRD deaths in the United States in 2000 (7). The annual percent change (APC) and its associated 95% confidence interval (CI) in age-adjusted mortality rates (AAMR) were calculated using the Joinpoint Regression Programme (Version 5.0.2, National Cancer Institute). This study was carried out to analyze annual changes in hypertension related ESRD mortality at the national level (19, 20). This approach utilizes log-linear regression models to detect meaningful alterations in age-adjusted mortality rates (AAMR) over time. The APCs were classified as increasing or decreasing if the slope reflecting changes in mortality differed substantially from zero, as assessed by two-tailed t-testing. A significance level of P<0.05 was used to establish statistical significance. Additionally, a sensitivity analysis was conducted for fatalities of hypertension related ESRD within the context of cardiovascular disease (CVD) when CVD (I00-I99) was identified as the primary cause of death, with hypertension related ESRD listed as a contributing cause of death.

## Results

Hypertension related ESRD caused a total of 721,511 deaths among adults (aged ≥ 35 years) between 1999 and 2020 (**Supplementary Table 1**). Of the 688,449 deaths where the location of death was recorded, 39% occurred at medical facilities, 25.6% took place in nursing homes/long-term care facilities, 6% at hospices, and 29.7% happened at home (**Supplementary Table 2**).

### Annual trends for hypertension related ESRD AAMR

**Figure 1:**
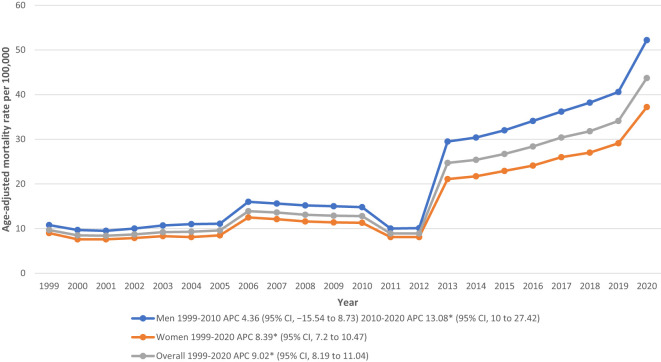
Overall and sex-stratified hypertension related ESRD AAMRs per 100,000 in adults in the United States, 1999 to 2020. * Indicates that the annual percentage change (APC) is significantly different from zero at α = 0.05. AAMR, age-adjusted mortality rate.

The overall AAMR for Hypertension related ESRD deaths in adults was 9.70 in 1999 and increased all the way up to 43.7 in 2020 (APC: 9.02; 95% CI: 8.19-11.04) (**Figure 1**; **Supplementary Tables 3, 4**). A more complicated trend was seen when deaths were confined to CVD as the underlying cause on sensitivity analysis. There was an increase in deaths from 1999 to 2005 (APC: 2.66; 95% CI: 1.86-3.68), then a decrease between 2005 and 2009 (APC: -4.24 95% CI: -6.15 to -2.66), followed by another increase from 2009 to 2015 (APC: 2.44; 95% CI: 1.23-3.67), and finally the steepest rise was seen between 2015 and 2020 (APC: 8.24; 95% CI: 7.4-9.47) (**Supplementary Figure 1**).

### Hypertension related ESRD AAMR stratified by sex

Men had consistently higher AAMRs than woman during the analyzed years (overall AAMR men: 23.1; 95% CI: 23.0-23.1; women: 16.4; 95% CI: 16.3-16.4). Starting with 1999, adult men had an AAMR of 10.8 (95% CI: 10.5-11) which rose to 14.8 in 2010 (APC: 4.36; 95% CI: -15.54 to 8.73). This rate reached its peak in 2020 with an AAMR of 52.2 (APC: 13.08; 95% CI: 10-27.42). Imitating this increasing trend, the Hypertension related ESRD AAMR for women climbed from 9 (95% CI: 8.8-9.2) in 1999 all the way up to 37.2 in 2020 (APC: 8.39; 95% CI: 7.2-10.47) (**Figure 1**; **Supplementary Tables 3, 4**).

### Hypertension related ESRD AAMR stratified by race/ethnicity

**Figure 2:**
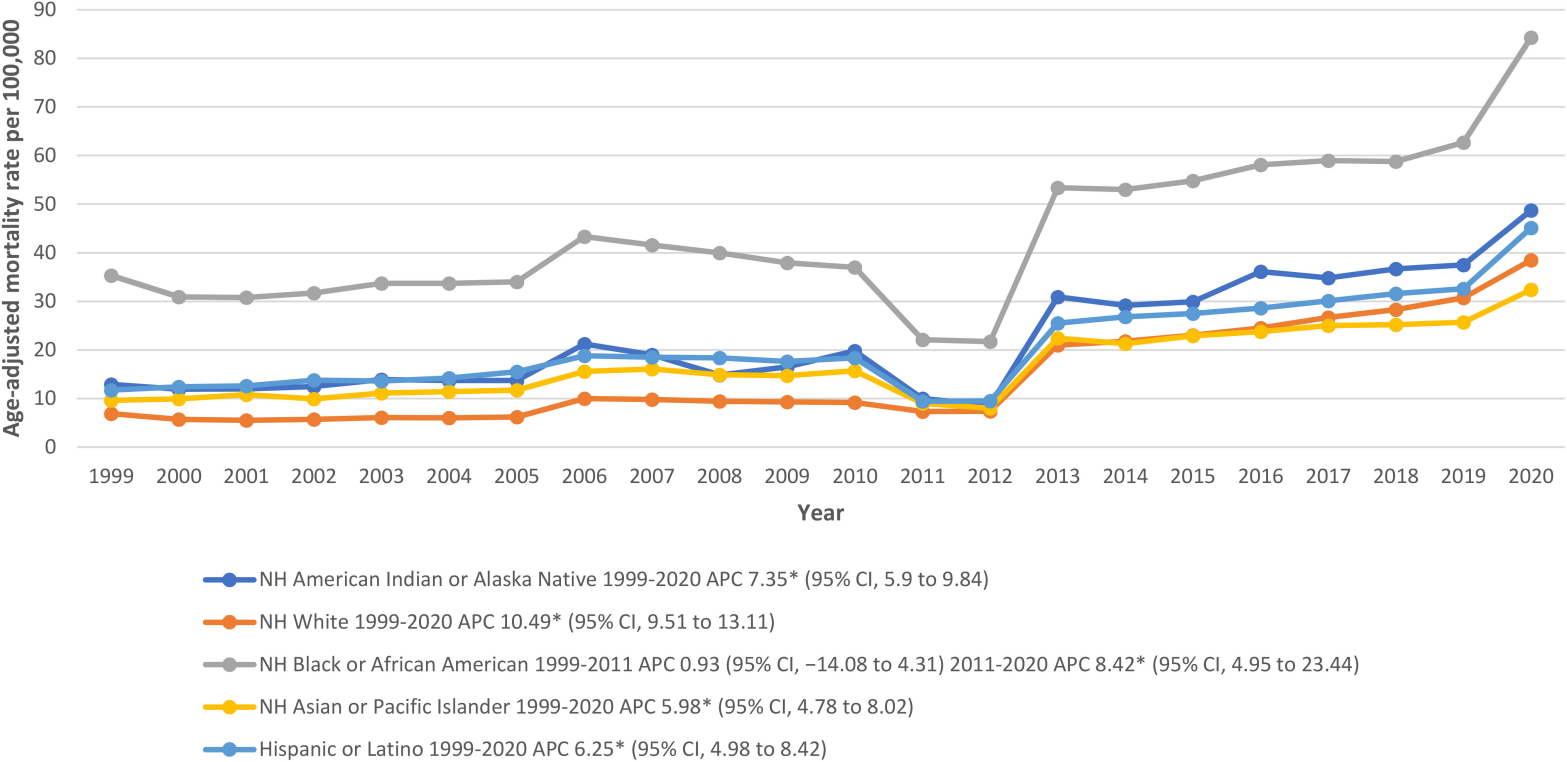
Hypertension related ESRD AAMRs per 100,000 stratified by race in adults in the United States, 1999 to 2020.* Indicates that the APC is significantly different from zero at α = 0.05. NH, non-Hispanic; other abbreviations as in **Figure 1**.

AAMR stratification according to race/ethnicity showed that rates were highest among NH Black or African American patients followed by NH American Indian or Alaska Native, Hispanic or Latino, NH Asian or Pacific Islander, and NH White populations (overall AAMR NH Black or African American: 45.7; 95% CI: 45.5-46; NH American Indian or Alaska Native: 24.7; 95% CI: 23.9-25.4; Hispanic or Latino: 23.4; 95% CI: 23.2-23.6; NH Asian or Pacific Islander: 19.3; 95% CI: 19-19.5; NH White: 15.4; 95% CI: 15.4-15.4). Death rates for NH Black or African Americans increased slightly from 1999-2011 (APC: 0.93; 95% CI: -14.08 to 4.31) and continued to hit its highest in 2020 (APC: 8.42; 95% CI: 4.95-23.44). NH American Indian or Alaska Native community also witnessed an increase in trend from 1999-2020 (APC: 7.35; 95% CI: 5.9-9.84). A similar escalation was seen in Hispanic or Latinos (APC: 6.25; 95% CI: 4.98-8.42), NH Asian or Pacific Islanders (APC: 5.98; 95% CI: 4.78-8.02), and NH White patients (APC: 10.49; 95% CI: 9.51-13.11) (**Figure 2**; **Supplementary Tables 3, 5**).

### Hypertension related ESRD AAMR stratified by geographic region

**Figure 3:**
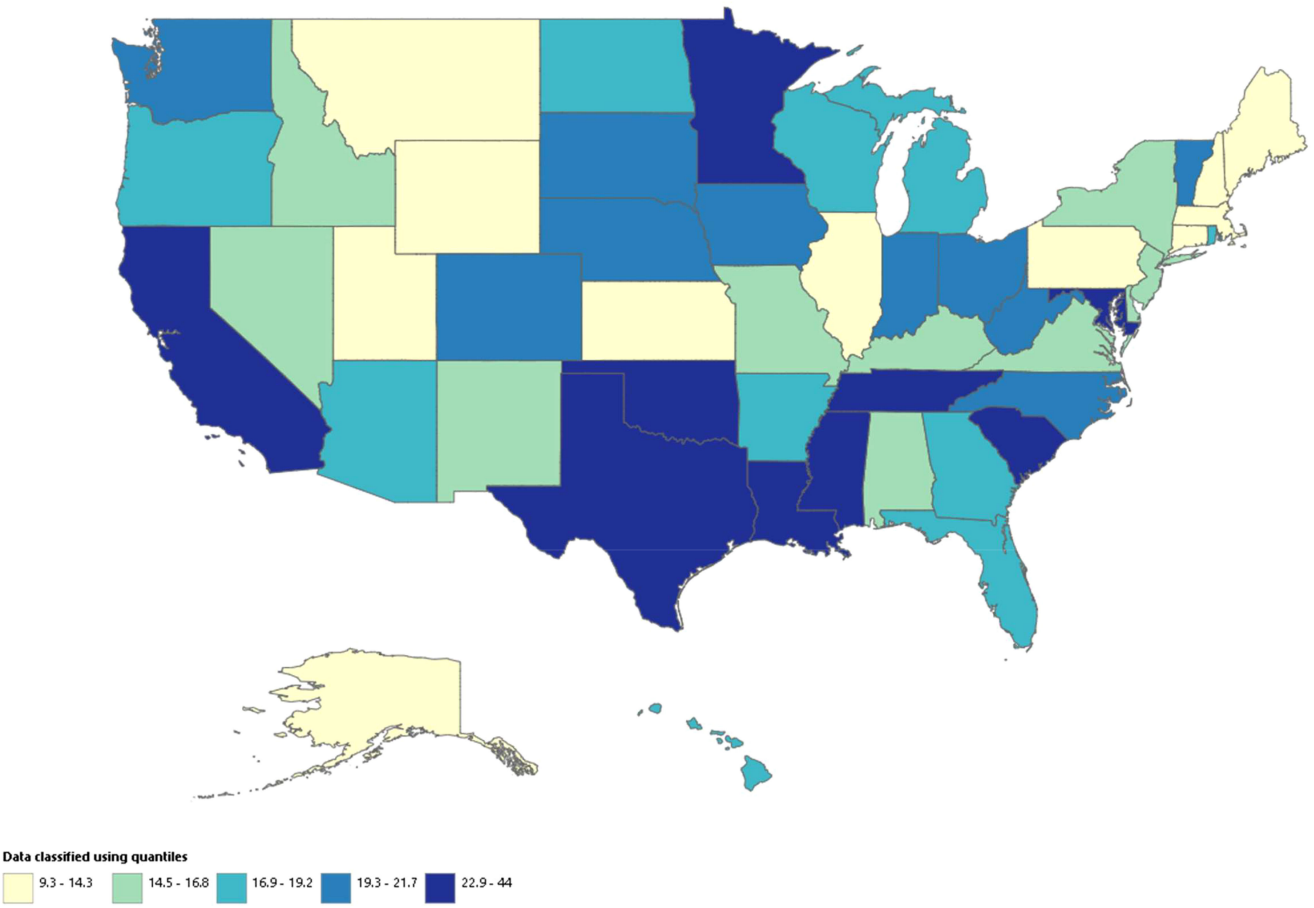
Hypertension related ESRD AAMRs per 100,000 stratified by state in adults in the United States, 1999 to 2020. Age-adjusted mortality rate per 100,000 among states, (9.3 – 44).

The states had a wide range of AAMRs starting from 9.3 (95% CI: 9-9.5) in Connecticut to 44 (95% CI: 42.4-45.6) in District of Columbia. States with death rates in the top 90th percentile (District of Columbia, Oklahoma, Mississippi, Tennessee, Texas, and South Carolina) had AAMRs more than double the ones found in the states in the lower 10th percentile (New Hampshire, Montana, Maine, Utah, Massachusetts, and Connecticut) (**Figure 3**; **Supplementary Table 6**).

We also analyzed the mortality rates according to census regions and found the highest AAMRs in the West (AAMR: 21.2; 95% CI: 21.1-21.3), followed by South (AAMR: 21; 95% CI: 20.9-21.1), Midwest (AAMR: 18.3; 95% CI: 18.2-18.4), and the Northeast (AAMR: 14.2; 95% CI: 14.1-14.3) (**Supplementary Table 7**).

**Figure 4:**
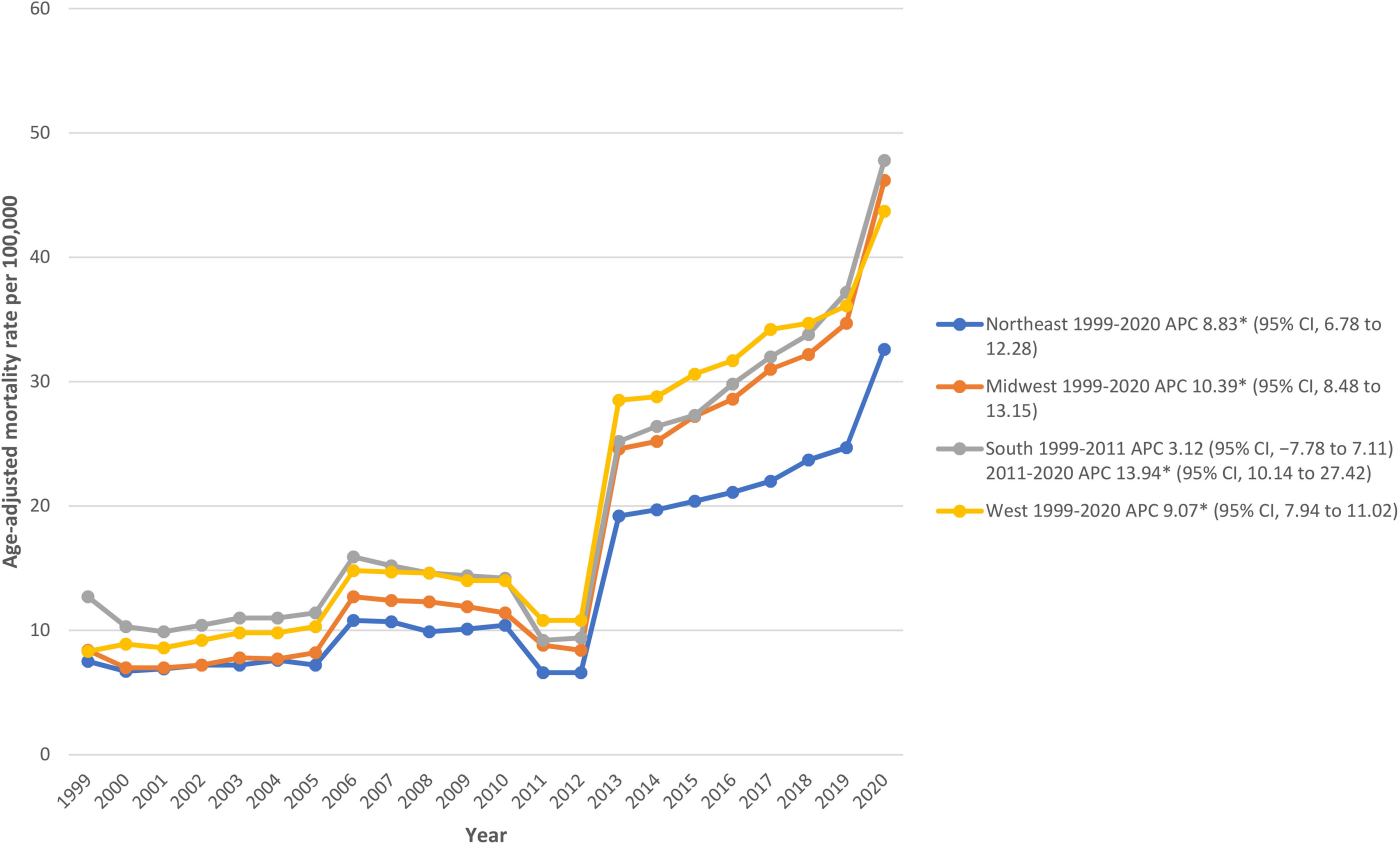
Hypertension related ESRD AAMRs per 100,000 stratified by census region in adults in the United States, 1999 to 2020.* Indicates that the APC is significantly different from zero at α = 0.05. Abbreviations as in **Figure 1**.

From 2000 to 2010, metropolitan areas had the lead in death rates which declined in 2011 and reached similar numbers to nonmetropolitan areas. From 2012 to 2020, AAMRs rose in both metropolitan and nonmetropolitan settings with nonmetropolitan ending the study period with the highest rates (Nonmetropolitan AAMR: 48.5; 95% CI: 47.7-49.2; Metropolitan AAMR: 42.8; 95% CI: 42.5-43.1). However, total AAMR for the study period was slightly higher in metropolitan areas (overall AAMRs: metropolitan 19.1; 95% CI: 19.1-19.2; nonmetropolitan: 19; 95% CI: 18.8-19.1). Overall, there was an increase in mortality rates in both metropolitan and non-metropolitan areas during the study period (Nonmetropolitan APC: 10.23; 95% CI: 8.96-12.52; Metropolitan APC: 8.34; 95% CI: 7.42-10.34) (**Figure 4**; **Supplementary Tables 3, 8**).

## Discussion

**Figure 5:**
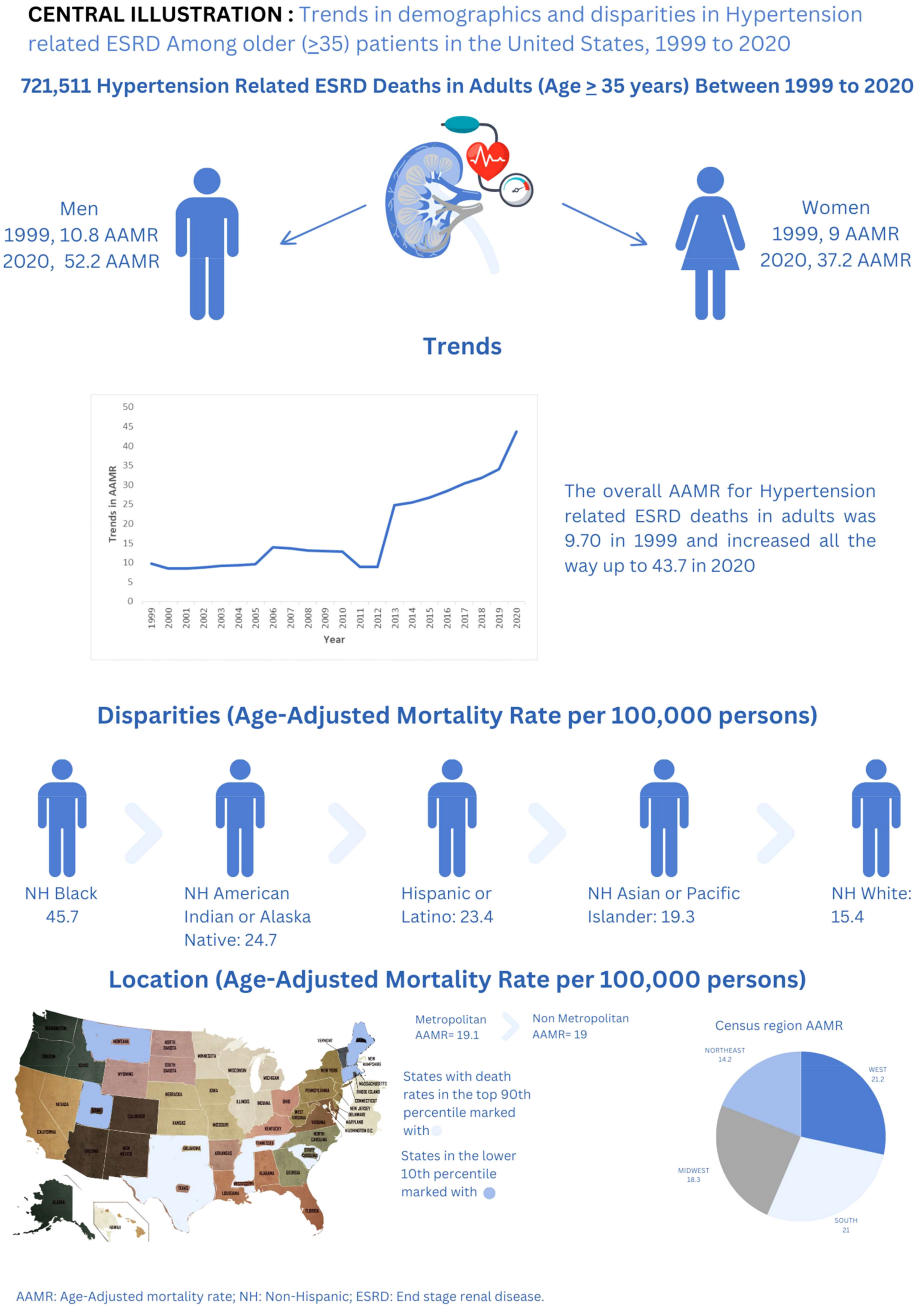
Central illustration.

The Centers for Disease Control and Prevention mortality data were thoroughly examined over a two-decade period, and numerous important findings were made. To start, our preliminary findings showed that death rates increased steadily from 1999 through 2020. However, after performing a sensitivity analysis, a more complex pattern became apparent. This analysis identified a unique trend in cardiovascular disease as the main cause of mortality in renal hypertensive illness. From 1999 to 2005, there was an initial tendency toward an increase in CVD-related mortality, which was followed by a drop until 2009. However, there was a rise in CVD-related mortality starting in 2009 and lasting until 2020. secondly male had a higher mortality rate and higher incline in mortality till 2020 as compared to females. third, NH blacks had higher AAMR followed by NH African Indian, Hispanics and NH Asians. fourthly, we noted substantial disparities among various regions of the United States. The West region exhibited the highest Age-Adjusted Mortality Rate (AAMR), followed by the South, Midwest, and Northeast. Furthermore, metropolitan areas demonstrated higher AAMR values until 2010, after which AAMR values gradually increased in both non-metropolitan areas and metropolitan areas eventually taking lead in non-metropolitan areas in the end. Additionally, AAMR values showed variation across different states, with states in the top 90th percentile having higher AAMR values compared to those in the 10th percentile. The ramifications of these findings for public health initiatives are considerable and obvious (**Figure 5**).

Renal hypertensive illness is a crippling ailment that significantly impairs those who are affected. Hypertension related ESRD which accounts for about 1-5% of instances of hypertension in the general population, especially in older people, is acknowledged as one of the main causes of high blood pressure (21).It also contributes to 20 to 40 percent of secondary hypertension cases and is a major factor in the emergence of resistant hypertension (22).The most common cause of this illness is atherosclerosis of the renal artery, which is frequently followed by fibromuscular dysplasia (21, 23). However, there are additional less frequent causes that can contribute to its occurrence, including trauma, vasculitis, antiphospholipid antibody syndrome (APLA), and renal artery dissection (24). This disorder has a few anomalies and consequences, including an increased risk of cardiovascular illnesses (22). Laboratory tests for this illness frequently demonstrate hypokalemia as well as elevated serum creatinine levels in response to ACE inhibitors (25).Stroke, ischemic renal disease, retinopathy, and pulmonary edema are just a few of the serious repercussions that could result from this illness if it is not properly handled (26).Some of its life-threatening complications encompass chronic kidney disease, chronic renal failure, and heart failure (27). Furthermore, it can accelerate the deterioration of kidney function in individuals already suffering from chronic kidney disease, leading to more rapid renal failure (28). Additionally, those who have this ailment frequently have a poor cardiovascular prognosis and are more likely to develop congestive heart failure, which can bring on a number of difficulties of its own (29, 30). Although there has reportedly been an increase in mortality rates recently, the evidence for this trend is still uncertain. Similarly, our trends have also increased in other disease that is related to this condition i.e., end stage renal disease (31).

In this condition, gender differences have also been noted. According to certain research, men are more likely than women to have hypertensive kidney disease, and women may also be more resilient to renal and cardiovascular consequences (32, 33). Men were more vulnerable to hypertension-induced alterations in renal function, according to a 7-year prospective research in a Chinese population (34). Males with pre-existing chronic renal illness deteriorated more quickly than females, according to a meta-analysis of 68 studies (35). However, in the case of hypertension related ESRD, a study found that the majority of patients who developed fibromuscular dysplasia with hypertension related ESRD and those who developed atherosclerotic renal disease with hypertension related ESRD were females (36).

In this situation, racial differences are clearly obvious. With an incidence rate that is seven times higher than that of Caucasians, African Americans are substantially more likely to develop hypertensive renal disease. In comparison to Caucasians, they are also more prone to cardiovascular issues (37). Additionally, African Americans are more likely to develop renal failure than other races, and those who have kidney disease in this group typically have a rapid reduction in renal function (38). However, it is important to note that Caucasians (39) made up the majority of those who acquired Hypertension related ESRD because of fibromuscular dysplasia. The regional differences are accounted for based on various factors, including different lifestyles, financial statuses, and the prevalence of various comorbid conditions. For instance, in certain areas of the western US, a lack of facilities contributes to an increase in cases of Hypertension related ESRD, along with more complex cases and higher mortality rates (37). The western US also exhibits a higher prevalence of obesity and diabetes, ultimately leading to an increase in cases of renal hypertensive disease and associated mortalities. Similarly, following the western regions, this condition has a high prevalence rate in the south due to similar risk factors; however, a sedentary lifestyle is also an important contributing factor toward mortality in Hypertension related ESRD (38).

### Limitations

It’s important to recognize a few constraints that have affected our analysis as we work to understand the variables affecting death rates. Even if they are significant, these limits offer important opportunities for further study and the improvement of our strategy. First, the lack of thorough clinical data, including specific biomarkers such as creatinine, GFR (Glomerular Filtration Rate), proteinuria, HbA1c, glycemia, LDL-C. Additionally, information about treatment modalities (i.e., blood pressure values, blood pressure variability), lab tests, or therapeutic interventions has been lacking and has hampered our efforts to understand the rise in fatalities. It’s important to note that the CDC WONDER database, our primary data source, doesn’t include clinical and laboratory characteristics data. As a result, detailed subgroup analysis for different prevalences of ESRD such as hypertensive/non-hypertensive, diabetic/non-diabetic, CKD/non-CKD, are not available. We are left with a knowledge gap because of this absence, making it impossible for us to identify the fundamental causes of the rise in death rates. Second, despite being vast, our database lacks crucial elements that can offer insightful information on the disease in question. Vital signs, GFR readings, renal scans, and biopsy results are noticeably lacking from the list of variables. Thirdly, the lack of information about medical therapy limits the scope of our analysis. Understanding the treatments used and how well they work may provide key insights into mortality patterns. Finally, socioeconomic information, a crucial driver of health outcomes, has not been considered in our analyses. Socioeconomic considerations have a significant impact on a person’s ability to receive healthcare and general well-being.

## Conclusions

Our results demonstrated an upward increase in AAMR from 1999 till 2020 in hypertensive renal disease. Men continued to have greater AAMRs than women, perpetuating gender inequities with Nh blacks dominating among other ethnicities. The western region of US showed higher AAMR values with metropolitan areas showing higher AAMR values initially till 2010 followed by a higher increase in AAMRs in non-metropolitan areas. The study emphasizes how urgent it is to address hypertension related ESRD as a serious public health issue that necessitates specialized interventions and healthcare strategies.

## Data availability statement

The original contributions presented in the study are included in the article/**Supplementary Material**. Further inquiries can be directed to the corresponding author.

## Ethics statement

Ethical approval was not required for the study involving humans in accordance with the local legislation and institutional requirements. Written informed consent to participate in this study was not required from the participants or the participants’ legal guardians/next of kin in accordance with the national legislation and the institutional requirements.

## Author contributions

AR: Conceptualization, Data curation, Formal Analysis, Investigation, Methodology, Project administration, Resources, Software, Supervision, Visualization, Writing – original draft, Writing – review & editing. SR: Conceptualization, Methodology, Project administration, Supervision, Validation, Visualization, Writing – original draft, Writing – review & editing. SB: Conceptualization, Formal Analysis, Methodology, Resources, Validation, Writing – original draft, Writing – review & editing. MS: Conceptualization, Formal Analysis, Methodology, Project administration, Visualization, Writing – original draft, Writing – review & editing. BA: Formal Analysis, Investigation, Resources, Software, Supervision, Validation, Visualization, Writing – original draft, Writing – review & editing. LK: Conceptualization, Investigation, Methodology, Validation, Visualization, Writing – original draft, Writing – review & editing.

## References

[1] Hypertensive Kidney Disease - an overview | ScienceDirect Topics (2023). Available at: https://www.sciencedirect.com/topics/medicine-and-dentistry/hypertensive-kidney-disease.

[2] Stompór T Perkowska-Ptasinska A. Hypertensive kidney disease: a true epidemic or rare disease? Pol Arch Intern Med (2020) 130(2):130–9. 10.20452/pamw.15150 31964856

[3] Tedla FM Brar A Browne R Brown C. Hypertension in chronic kidney disease: Navigating the evidence. Int J Hypertens (2011) 2011. 10.4061/2011/132405 PMC3124254 21747971

[4] Hypertension induced by drugs and other substances (2023). 7777726

[5] Dunea G Arruda JAL Bakir AA Share DS Smith EC. Role of cocaine in end-stage renal disease in some hypertensive african americans. Am J Nephrol (1995) 15(1):5–9. doi: 10.1159/000168794 7872365

[6] Torres PA Helmstetter JA Kaye AM Kaye AD. Rhabdomyolysis: pathogenesis, diagnosis, and treatment. Ochsner J (2015) 15(1):58. 25829882 PMC4365849

[7] Gordon Walker W. Hypertension-related renal injury: a major contributor to end-stage renal disease. Am J Kidney Dis (1993) 22(1):164–73. 10.1016/s0272-6386(12)70183-x 8322780

[8] Congestive heart failure and renal complications.

[9] Schlessinger SD Tankersley MR Curtis JJ. Clinical documentation of end-stage renal disease due to hypertension. Am J Kidney Diseases. (1994) 23(5):655–60. 10.1016/s0272-6386(12)70275-5 8172207

[10] Kidney disease in Native Americans.

[11] Gilbertson DT Liu J Xue JL Louis TA Solid CA Ebben JP. Projecting the number of patients with end-stage renal disease in the United States to the year 2015. J Am Soc Nephrol (2005) 16(12):3736–41. 10.1681/ASN.2005010112 16267160

[12] Ren Y Zhang H. The trend of hypertension-related chronic kidney disease from 1990 to 2019 and its predictions over 25 years: An analysis of the Global Burden of Disease Study 2019. medRxiv (2023). doi: 10.1101/2023.03.21.23287527v1 37542001

[13] Mailloux LU Napolitano B Bellucci AG Vernace M Wilkes BM Mossey RT. Renal vascular disease causing end-stage renal disease, incidence, clinical correlates, and outcomes: a 20-year clinical experience. Am J Kidney Dis (1994) 24(4):622–9. 10.1016/s0272-6386(12)80223-x 7942820

[14] Bakris GL Williams M Dworkin L Elliott WJ Epstein M Toto R. Preserving renal function in adults with hypertension and diabetes: a consensus approach. National Kidney Foundation Hypertension and Diabetes Executive Committees Working Group. Am J Kidney Dis (2000) 36(3):646–61. 10.1053/ajkd.2000.16225 10977801

[15] Tierney WM McDonald CJ Luft FC. Renal disease in hypertensive adults: effect of race and type II diabetes mellitus. Am J Kidney Dis (1989) 13(6):485–93. 10.1016/s0272-6386(89)80006-x 2729268

[16] Multiple cause of death, 1999-2020 results (2023). Available at: https://wonder.cdc.gov/controller/datarequest/D77;jsessionid=CBB7708644CDF18DCBA4BAB1EEB1.

[17] Aggarwal R Chiu N Loccoh EC Kazi DS Yeh RW Wadhera RK. Rural-urban disparities: diabetes, hypertension, heart disease, and stroke mortality among black and white adults, 1999–2018. J Am Coll Cardiol (2021) 77(11):1480. 33736831 10.1016/j.jacc.2021.01.032 PMC8210746

[18] 2013 NCHS urban-rural classification scheme for counties (2023). 24776070

[19] Joinpoint — Joinpoint help system (2023). Available at: https://surveillance.cancer.gov/help/joinpoint.

[20] Age standardization of death rates: implementation of the year 2000 standard (2023). 9796247

[21] Herrmann SM Textor SC. Renovascular hypertension. Endocrinol Metab Clin North Am (2019) 48(4):765–78. 10.1016/j.ecl.2019.08.007 PMC7184322 31655775

[22] Mannemuddhu SS Ojeda JC Yadav A. Renovascular hypertension. Primary care - clinics in office practice (2020). Available at: http://www.primarycare.theclinics.com/article/S0095454320300634/fulltext. 10.1016/j.pop.2020.08.009 33121633

[23] Boutari C Georgianou E Sachinidis A Katsimardou A Christou K Piperidou A. Renovascular hypertension: novel insights. Curr Hypertens Rev (2020) 16(1):24–9. 10.2174/1573402115666190416153321 31038069

[24] Textor SC Misra S Oderich GS. Percutaneous revascularization for ischemic nephropathy: the past, present, and future. Kidney Int (2013) 83(1):28–40. 23151953 10.1038/ki.2012.363 PMC3532568

[25] Taler SJ. Initial treatment of hypertension. N Engl J Med (2018) 378(7):636–44. 10.1056/NEJMcp1613481 29443671

[26] Nair R Vaqar S. Renovascular hypertension. StatPearls (2023). Available at: https://www.ncbi.nlm.nih.gov/books/NBK551587/. 31869068

[27] Burnei G Burnei A Hodorogea D Drǎghici I Georgescu I Vlad C. Diagnosis and complications of renovascular hypertension in children: literature data and clinical observations. J Med Life (2009) 2(1):18–28. 20108487 PMC5051477

[28] Krzesinski JM Cohen EP. Hypertension and the kidney. Acta Clin Belg (2007) 62(1):5–14. 17451140 10.1179/acb.2007.002

[29] Conlon PJ Athirakul K Kovalik E Schwab SJ Crowley J Stack R. Survival in renal vascular disease. J Am Soc Nephrol (1998) 9(2):252–6. 10.1681/ASN.V92252 9527401

[30] De Silva R Nikitin NP Bhandari S Nicholson A Clark AL Cleland JGF. Atherosclerotic renovascular disease in chronic heart failure: should we intervene? Eur Heart J (2005) 26(16):1596–605. 10.1093/eurheartj/ehi304 15919719

[31] Albertus P Morgenstern H Robinson B Saran R. Risk of ESRD in the United States. Am J Kidney Dis (2016) 68(6):862–72. 10.1053/j.ajkd.2016.05.030 PMC5123906 27578184

[32] Gillis EE Sullivan JC. Sex differences in hypertension: recent advances. Hypertension (2016) 68(6):1322–7. 10.1161/HYPERTENSIONAHA.116.06602 PMC5159215 27777357

[33] Baiardi G Macova M Armando I Ando H Tyurmin D Saavedra JM. Estrogen upregulates renal angiotensin II AT1 and AT2 receptors in the rat. Regul Pept (2005) 124(1–3):7–17. 15544836 10.1016/j.regpep.2004.06.021

[34] Wang Q Xie D Xu X Qin X Tang G Wang B. Blood pressure and renal function decline: a 7-year prospective cohort study in middle-aged rural Chinese men and women. J Hypertens (2015) 33(1):136–43. 10.1097/HJH.0000000000000360 25255396

[35] Neugarten J Acharya A Silbiger SR. Effect of gender on the progression of nondiabetic renal disease: a meta-analysis. J Am Soc Nephrol (2000) 11(2):319–29. 10.1681/ASN.V112319 10665939

[36] Nikolić M Kuzmanić D Jelaković B. Clinical characteristics of patients with renovascular hypertension. (2006). europepmc.orgM Nikolić, D Kuzmanić, B Jelaković, M Laganović, TZ Vrkić, BP NikolićLijecnicki Vjesnik, 2006•europepmc.org. 17212204

[37] Lea JP. Racial disparities in hypertensive cardiovascular disease and chronic kidney disease. FASEB J (2010) 24(S1).

[38] Fogo AB. Hypertensive risk factors in kidney disease in African Americans. Kidney Int Suppl (2003) 63(83). 10.1046/j.1523-1755.63.s83.5.x 12864869

[39] Rana MN Al-Kindi SG. Prevalence and manifestations of diagnosed fibromuscular dysplasia by sex and race: Analysis of >4500 FMD cases in the United States. Heart Lung (2021) 50(1):168–73. 10.1016/j.hrtlng.2020.09.022 33069453

