# Supplementary material for: Temporal trends in hypertension related end stage renal disease mortality rates: an analysis of gender, race/ethnicity, and geographic disparities in the United States
Source: Front Nephrol. 2024 Jan 15;3:1339312. doi: 10.3389/fneph.2023.1339312 (PMC10823365; doi:10.3389/fneph.2023.1339312)
Supplement: Supplementary file 1 [file DataSheet_1.docx]

**Supplemental Table 1** Hypertension-related ESRD Deaths, Stratified by Sex and Race, in Adults in the United States, 1999 to 2020.

| **Deaths** | | | | | | | | | |
| --- | --- | --- | --- | --- | --- | --- | --- | --- | --- |
| **Year** | **Overall** | **Women** | **Men** | **NH White** | **NH Black or African American** | **NH Asian or Pacific Islander** | **NH American Indian or Alaska Native** | **Hispanic or Latino** | **Population** |
| 1999 | 13500 | 7657 | 5843 | 8097 | 4204 | 289 | 77 | 795 | 140230363 |
| 2000 | 11956 | 6395 | 5561 | 6755 | 3825 | 326 | 80 | 922 | 142092916 |
| 2001 | 11985 | 6439 | 5546 | 6581 | 3900 | 388 | 79 | 993 | 144833606 |
| 2002 | 12767 | 6745 | 6022 | 6955 | 4109 | 382 | 91 | 1189 | 146858382 |
| 2003 | 13709 | 7178 | 6531 | 7423 | 4470 | 453 | 100 | 1213 | 148846634 |
| 2004 | 14032 | 7128 | 6904 | 7484 | 4563 | 496 | 108 | 1342 | 150938828 |
| 2005 | 14752 | 7605 | 7147 | 7805 | 4726 | 532 | 107 | 1541 | 153292737 |
| 2006 | 21812 | 11588 | 10224 | 12895 | 6044 | 746 | 155 | 1926 | 155624180 |
| 2007 | 21707 | 11473 | 10234 | 12798 | 5966 | 796 | 152 | 1967 | 157690314 |
| 2008 | 21506 | 11260 | 10246 | 12580 | 5907 | 776 | 125 | 2067 | 159587617 |
| 2009 | 21497 | 11193 | 10304 | 12654 | 5745 | 809 | 140 | 2089 | 161383674 |
| 2010 | 21712 | 11277 | 10435 | 12593 | 5742 | 900 | 175 | 2258 | 162828035 |
| 2011 | 15580 | 8546 | 7034 | 10354 | 3438 | 533 | 81 | 1146 | 164802438 |
| 2012 | 16037 | 8765 | 7272 | 10702 | 3469 | 510 | 79 | 1236 | 166516716 |
| 2013 | 45170 | 23317 | 21853 | 31043 | 8715 | 1549 | 283 | 3474 | 168240727 |
| 2014 | 47565 | 24401 | 23164 | 32669 | 8975 | 1599 | 284 | 3910 | 170292776 |
| 2015 | 51172 | 26170 | 25002 | 35018 | 9561 | 1833 | 309 | 4266 | 172416615 |
| 2016 | 55402 | 28022 | 27380 | 37714 | 10450 | 2008 | 392 | 4664 | 173964174 |
| 2017 | 60767 | 30939 | 29828 | 41810 | 10941 | 2265 | 396 | 5189 | 176104659 |
| 2018 | 65024 | 32625 | 32399 | 45050 | 11253 | 2410 | 446 | 5685 | 177613416 |
| 2019 | 71099 | 35827 | 35272 | 49518 | 12312 | 2592 | 460 | 6066 | 179040846 |
| 2020 | 92760 | 46337 | 46423 | 62687 | 16987 | 3447 | 650 | 8821 | 180565367 |
| **Total** | **721,511** | **370,887** | **350,624** | **471,185** | **155,302** | **25,639** | **4,769** | **62,759** | **3,553,765,020** |
| NH, non-Hispanic. |  |  |  |  |  |  |  |  |  |

**Supplemental Table 2** Hypertension-related ESRD Mortality, Stratified by Place of Death in Adults in the United States, 1999 to 2020.

| **Deaths** | | | | |  |
| --- | --- | --- | --- | --- | --- |
| **Year** | **Medical Facility** | **Nursing Home/Long-term Care Facility** | **Hospices** | **Home** |  |
|  |  |  |  |  |  |
| 1999 | 7,458 | 3292 | Missing | 2319 |  |
| 2000 | 7,485 | 2177 | Missing | 1888 |  |
| 2001 | 7,338 | 2261 | Missing | 1904 |  |
| 2002 | 7,534 | 2505 | Missing | 2136 |  |
| 2003 | 8,020 | 2633 | 28 | 2340 |  |
| 2004 | 8,002 | 2703 | 45 | 2596 |  |
| 2005 | 8,298 | 2815 | 273 | 2761 |  |
| 2006 | 10,882 | 5125 | 567 | 4377 |  |
| 2007 | 10,435 | 5090 | 804 | 4526 |  |
| 2008 | 9,991 | 4901 | 870 | 4565 |  |
| 2009 | 9,564 | 4907 | 996 | 4724 |  |
| 2010 | 9,536 | 4939 | 1206 | 5143 |  |
| 2011 | 5,299 | 4264 | 964 | 4303 |  |
| 2012 | 5,038 | 4357 | 1064 | 4768 |  |
| 2013 | 15,648 | 12179 | 2390 | 12761 |  |
| 2014 | 16,112 | 12878 | 2987 | 13593 |  |
| 2015 | 16,789 | 13812 | 3547 | 14874 |  |
| 2016 | 17,659 | 14289 | 4171 | 16878 |  |
| 2017 | 18,584 | 15819 | 4604 | 18916 |  |
| 2018 | 19,176 | 16821 | 4914 | 21110 |  |
| 2019 | 20,752 | 17650 | 5589 | 23759 |  |
| 2020 | 27,467 | 20649 | 6125 | 33931 |  |
| **Total** | **267,067** | **176,066** | **41,144** | **204,172** |  |

**Supplemental Table 3** Annual percent change of Hypertension-related ESRD Age-Adjusted Mortality Rates per 100,000 in Adults in the United States, 1999 to 2020

|  |  |
| --- | --- |
| **Year Interval** | **APC (95% CI)** |
| **Overall** | |
| 1999-2020 | 9.02* (8.19 to 11.04) |
| **Men** | |
| 1999-2010 | 4.36 (-15.54 to 8.73) |
| 2010-2020 | 13.08* (10 to 27.42) |
| **Women** | |
| 1999-2020 | 8.39* (7.2 to 10.47) |
| **NH White** | |
| 1999-2020 | 10.49* (9.51 to 13.11) |
| **NH Black or African American** | |
| 1999-2011 | 0.93 (−14.08 to 4.31) |
| 2011-2020 | 8.42* (4.95 to 23.44) |
| **NH American Indian or Alaska Native** | |
| 1999-2020 | 7.35* (5.9 to 9.84) |
| **Hispanic or Latino** | |
| 1999-2020 | 6.25* (4.98 to 8.42) |
| **NH Asian or Pacific Islander** | |
| 1999-2020 | 5.98* (4.78 to 8.02) |
| **Nonmetropolitan areas** | |
| 1999-2020 | 10.23* (8.96 to12.52) |
| **Metropolitan area** | |
| 1999-2020 | 8.34* (7.42 to 10.34) |
| **Northeast region** | |
| 1999-2020 | 8.83* (6.78 to 12.28) |
| **Midwest region** | |
| 1999-2020 | 10.39* (8.48 to 13.15) |
| **South region** | |
| 1999-2011 | 3.12 (-7.78 to 7.11) |
| 2011-2020 | 13.94* (10.14 to 27.42) |
| **West region** | |
| 1999-2020 | 9.07* (7.94 to 11.02) |

APC = annual percent change; NH = non-Hispanic; * Indicates that the annual percentage change (APC) is significantly different from zero at α = 0.05. AAMR = age-adjusted mortality rate.

**Supplemental Table 4** Overall and Sex‐Stratified Hypertension-related ESRD Age-Adjusted Mortality Rates per 100,000 in Adults in the United States, 1999 to 2020.

| **Age-Adjusted Rate (95% CI)** | | | |
| --- | --- | --- | --- |
| **Year** | **Men** | **Women** | **Overall** |
| 1999 | 10.8 (10.5-11) | 9 (8.8-9.2) | 9.7 (9.5-9.8) |
| 2000 | 9.7 (9.5-10) | 7.6 (7.4-7.8) | 8.5 (8.3-8.6) |
| 2001 | 9.5 (9.3-9.8) | 7.6 (7.4-7.8) | 8.4 (8.2-8.5) |
| 2002 | 10 (9.8-10.3) | 7.9 (7.7-8) | 8.7 (8.6-8.9) |
| 2003 | 10.7 (10.4-10.9) | 8.3 (8.1-8.5) | 9.2 (9.1-9.4) |
| 2004 | 11 (10.8-11.3) | 8.1 (7.9-8.3) | 9.3 (9.2-9.5) |
| 2005 | 11.1 (10.9-11.4) | 8.5 (8.3-8.7) | 9.6 (9.4-9.7) |
| 2006 | 16 (15.7-16.3) | 12.5 (12.2-12.7) | 13.9 (13.7-14.1) |
| 2007 | 15.6 (15.3-15.9) | 12.1 (11.9-12.3) | 13.6 (13.4-13.7) |
| 2008 | 15.2 (14.9-15.5) | 11.6 (11.4-11.9) | 13.1 (13-13.3) |
| 2009 | 15 (14.7-15.3) | 11.4 (11.2-11.6) | 12.9 (12.7-13.1) |
| 2010 | 14.8 (14.5-15.1) | 11.3 (11.1-11.5) | 12.8 (12.7-13) |
| 2011 | 10 (9.8-10.3) | 8.1 (7.9-8.3) | 8.9 (8.8-9) |
| 2012 | 10.1 (9.8-10.3) | 8.1 (7.9-8.2) | 8.9 (8.8-9.1) |
| 2013 | 29.5 (29.1-29.9) | 21.1 (20.9-21.4) | 24.7 (24.4-24.9) |
| 2014 | 30.4 (30-30.8) | 21.7 (21.4-22) | 25.4 (25.1-25.6) |
| 2015 | 32 (31.6-32.4) | 22.9 (22.6-23.2) | 26.7 (26.4-26.9) |
| 2016 | 34.1 (33.7-34.5) | 24.1 (23.8-24.4) | 28.4 (28.1-28.6) |
| 2017 | 36.2 (35.8-36.7) | 26 (25.7-26.3) | 30.4 (30.2-30.7) |
| 2018 | 38.2 (37.8-38.7) | 27 (26.7-27.3) | 31.8 (31.5-32) |
| 2019 | 40.6 (40.2-41) | 29.1 (28.8-29.4) | 34.1 (33.8-34.3) |
| 2020 | 52.2 (51.7-52.7) | 37.2 (36.8-37.5) | 43.7 (43.5-44) |
| Total | 23.1 (23-23.1) | 16.4 (16.3-16.4) | 19.1 (19.1-19.1) |

**Supplemental Table 5** Hypertension-related ESRD Age-Adjusted Mortality Rates per 100,000, Stratified by Race in Adults in the United States, 1999 to 2020.

| **Age-Adjusted Rate (95% CI)** | | | | | |
| --- | --- | --- | --- | --- | --- |
| **Year** | **NH White** | **NH Black or African American** | **NH American Indian or Alaska Native** | **Hispanic or Latino** | **NH Asian or Pacific Islander** |
| 1999 | 6.9 (6.7-7) | 35.3 (34.2-36.4) | 12.9 (10-16.3) | 11.7 (10.8-12.5) | 9.6 (8.4-10.7) |
| 2000 | 5.7 (5.6-5.8) | 30.9 (30-31.9) | 11.9 (9.3-15) | 12.4 (11.5-13.2) | 9.9 (8.8-11.1) |
| 2001 | 5.5 (5.4-5.6) | 30.8 (29.8-31.7) | 12 (9.4-15.2) | 12.6 (11.8-13.4) | 10.8 (9.7-11.9) |
| 2002 | 5.7 (5.6-5.9) | 31.7 (30.8-32.7) | 12.5 (9.9-15.6) | 13.8 (13-14.6) | 9.9 (8.9-10.9) |
| 2003 | 6.1 (5.9-6.2) | 33.7 (32.6-34.7) | 13.9 (11-16.8) | 13.6 (12.8-14.4) | 11.1 (10.1-12.2) |
| 2004 | 6 (5.9-6.2) | 33.7 (32.7-34.7) | 13.7 (10.9-16.4) | 14.2 (13.4-15) | 11.4 (10.3-12.4) |
| 2005 | 6.2 (6.1-6.3) | 34 (33-35) | 13.7 (10.9-16.5) | 15.5 (14.7-16.3) | 11.7 (10.6-12.7) |
| 2006 | 10 (9.8-10.2) | 43.3 (42.2-44.4) | 21.2 (17.6-24.8) | 18.8 (17.9-19.6) | 15.6 (14.4-16.7) |
| 2007 | 9.8 (9.6-9.9) | 41.6 (40.5-42.7) | 19 (15.8-22.3) | 18.5 (17.6-19.3) | 16.1 (15-17.3) |
| 2008 | 9.4 (9.3-9.6) | 40 (39-41.1) | 14.8 (12-17.6) | 18.4 (17.5-19.2) | 14.9 (13.8-15.9) |
| 2009 | 9.3 (9.2-9.5) | 37.9 (36.9-39) | 16.5 (13.6-19.5) | 17.6 (16.8-18.3) | 14.7 (13.7-15.7) |
| 2010 | 9.2 (9.1-9.4) | 37 (36-38) | 19.8 (16.7-23) | 18.4 (17.6-19.2) | 15.7 (14.7-16.8) |
| 2011 | 7.3 (7.1-7.4) | 22.1 (21.4-22.9) | 10 (7.8-12.6) | 9.4 (8.8-9.9) | 9 (8.2-9.7) |
| 2012 | 7.4 (7.2-7.5) | 21.7 (20.9-22.4) | 8.6 (6.7-10.9) | 9.5 (9-10.1) | 8 (7.3-8.7) |
| 2013 | 21 (20.8-21.3) | 53.4 (52.2-54.5) | 30.9 (27.1-34.7) | 25.5 (24.6-26.4) | 22.4 (21.3-23.5) |
| 2014 | 21.8 (21.6-22.1) | 53 (51.9-54.1) | 29.2 (25.6-32.7) | 26.8 (25.9-27.6) | 21.3 (20.2-22.3) |
| 2015 | 23 (22.8-23.3) | 54.8 (53.6-55.9) | 29.9 (26.4-33.3) | 27.5 (26.6-28.3) | 22.9 (21.8-23.9) |
| 2016 | 24.5 (24.2-24.7) | 58.1 (57-59.3) | 36.1 (32.4-39.8) | 28.6 (27.7-29.4) | 23.8 (22.8-24.9) |
| 2017 | 26.7 (26.5-27) | 59 (57.9-60.2) | 34.8 (31.3-38.4) | 30.1 (29.2-30.9) | 25 (24-26.1) |
| 2018 | 28.3 (28-28.6) | 58.8 (57.6-59.9) | 36.7 (33.2-40.3) | 31.6 (30.7-32.4) | 25.2 (24.2-26.2) |
| 2019 | 30.7 (30.4-30.9) | 62.7 (61.5-63.8) | 37.5 (34-41) | 32.6 (31.7-33.4) | 25.7 (24.7-26.7) |
| 2020 | 38.5 (38.1-38.8) | 84.3 (83-85.6) | 48.7 (44.8-52.6) | 45.1 (44.1-46.1) | 32.4 (31.3-33.5) |
| **Total** | 15.4 (15.4-15.4) | 45.7 (45.5-46) | 24.7 (23.9-25.4) | 23.4 (23.2-23.6) | 19.3 (19.0-19.5) |
| NH = non-Hispanic. |  |  |  |  |  |

**Supplemental Table 6** Hypertension-related ESRD Age-Adjusted Mortality Rates per 100,000, Stratified by States in Adults in the United States, 1999 to 2020.

| **State** | **Age-Adjusted Rate (95% CI)** |
| --- | --- |
| Alabama | 15.4 (15.1-15.8) |
| Alaska | 12.7 (11.6-13.8) |
| Arizona | 19.2 (18.9-19.5) |
| Arkansas | 18.1 (17.7-18.5) |
| California | 24.5 (24.3-24.6) |
| Colorado | 19.7 (19.4-20.1) |
| Connecticut | 9.3 (9-9.5) |
| Delaware | 15.4 (14.7-16.1) |
| District of Columbia | 44 (42.4-45.6) |
| Florida | 16.9 (16.8-17.1) |
| Georgia | 17.4 (17.2-17.7) |
| Hawaii | 18.4 (17.8-19) |
| Idaho | 16.8 (16.2-17.4) |
| Illinois | 14.3 (14.1-14.5) |
| Indiana | 20.9 (20.6-21.2) |
| Iowa | 19.3 (18.9-19.7) |
| Kansas | 12.4 (12-12.8) |
| Kentucky | 16.6 (16.3-17) |
| Louisiana | 23.1 (22.7-23.5) |
| Maine | 10.7 (10.3-11.2) |
| Maryland | 22.9 (22.5-23.2) |
| Massachusetts | 9.6 (9.4-9.8) |
| Michigan | 17.1 (16.8-17.3) |
| Minnesota | 23.3 (22.9-23.6) |
| Mississippi | 29.5 (29-30.1) |
| Missouri | 15.5 (15.3-15.8) |
| Montana | 11.7 (11.2-12.3) |
| Nebraska | 21.2 (20.7-21.8) |
| Nevada | 15 (14.5-15.5) |
| New Hampshire | 12.1 (11.5-12.6) |
| New Jersey | 14.5 (14.3-14.7) |
| New Mexico | 14.7 (14.2-15.2) |
| New York | 16.7 (16.5-16.8) |
| North Carolina | 19.7 (19.4-20) |
| North Dakota | 18.4 (17.5-19.2) |
| Ohio | 21.3 (21-21.5) |
| Oklahoma | 31.4 (30.9-31.9) |
| Oregon | 19 (18.6-19.4) |
| Pennsylvania | 14 (13.8-14.2) |
| Rhode Island | 17.9 (17.3-18.6) |
| South Carolina | 25.3 (24.8-25.7) |
| South Dakota | 20.9 (20.1-21.7) |
| Tennessee | 26.4 (26.1-26.8) |
| Texas | 25.9 (25.7-26.1) |
| Utah | 10.4 (10-10.9) |
| Vermont | 21.7 (20.7-22.7) |
| Virginia | 15.3 (15.1-15.6) |
| Washington | 20.4 (20.1-20.7) |
| West Virginia | 19.5 (19-20.1) |
| Wisconsin | 18.2 (17.9-18.5) |
| Wyoming | 13 (12.1-13.9) |

**Supplemental Table 7** Hypertension-related ESRD Age-Adjusted Mortality Rates per 100,000, Stratified by Census Region in Adults in the United States, 1999 to 2020.

| **Census Region** | **Year** | **Age-Adjusted Rate (95% CI)** |
| --- | --- | --- |
| Northeast | 1999 | 7.5 (7.2-7.8) |
| Northeast | 2000 | 6.7 (6.4-7) |
| Northeast | 2001 | 6.9 (6.6-7.2) |
| Northeast | 2002 | 7.2 (6.9-7.5) |
| Northeast | 2003 | 7.2 (6.9-7.5) |
| Northeast | 2004 | 7.6 (7.3-7.9) |
| Northeast | 2005 | 7.2 (6.9-7.5) |
| Northeast | 2006 | 10.8 (10.4-11.1) |
| Northeast | 2007 | 10.7 (10.3-11.1) |
| Northeast | 2008 | 9.9 (9.6-10.3) |
| Northeast | 2009 | 10.1 (9.7-10.4) |
| Northeast | 2010 | 10.4 (10-10.7) |
| Northeast | 2011 | 6.6 (6.3-6.9) |
| Northeast | 2012 | 6.6 (6.3-6.9) |
| Northeast | 2013 | 19.2 (18.8-19.7) |
| Northeast | 2014 | 19.7 (19.3-20.2) |
| Northeast | 2015 | 20.4 (19.9-20.8) |
| Northeast | 2016 | 21.1 (20.6-21.6) |
| Northeast | 2017 | 22 (21.5-22.5) |
| Northeast | 2018 | 23.7 (23.2-24.2) |
| Northeast | 2019 | 24.7 (24.2-25.2) |
| Northeast | 2020 | 32.6 (32-33.1) |
| Northeast | **Total** | 14.2 (14.1-14.3) |
| Midwest | 1999 | 8.4 (8.1-8.7) |
| Midwest | 2000 | 7 (6.7-7.3) |
| Midwest | 2001 | 7 (6.7-7.3) |
| Midwest | 2002 | 7.2 (6.9-7.5) |
| Midwest | 2003 | 7.8 (7.5-8.1) |
| Midwest | 2004 | 7.7 (7.4-8) |
| Midwest | 2005 | 8.2 (7.9-8.5) |
| Midwest | 2006 | 12.7 (12.3-13) |
| Midwest | 2007 | 12.4 (12.1-12.8) |
| Midwest | 2008 | 12.3 (11.9-12.6) |
| Midwest | 2009 | 11.9 (11.6-12.3) |
| Midwest | 2010 | 11.4 (11.1-11.8) |
| Midwest | 2011 | 8.8 (8.5-9.1) |
| Midwest | 2012 | 8.4 (8.1-8.7) |
| Midwest | 2013 | 24.6 (24.1-25.1) |
| Midwest | 2014 | 25.2 (24.7-25.6) |
| Midwest | 2015 | 27.2 (26.7-27.7) |
| Midwest | 2016 | 28.6 (28.1-29.1) |
| Midwest | 2017 | 31 (30.5-31.6) |
| Midwest | 2018 | 32.2 (31.7-32.8) |
| Midwest | 2019 | 34.7 (34.1-35.2) |
| Midwest | 2020 | 46.2 (45.6-46.8) |
| Midwest | **Total** | 18.3 (18.2-18.4) |
| South | 1999 | 12.7 (12.4-13) |
| South | 2000 | 10.3 (10-10.5) |
| South | 2001 | 9.9 (9.7-10.2) |
| South | 2002 | 10.4 (10.2-10.7) |
| South | 2003 | 11 (10.7-11.2) |
| South | 2004 | 11 (10.7-11.3) |
| South | 2005 | 11.4 (11.1-11.7) |
| South | 2006 | 15.9 (15.5-16.2) |
| South | 2007 | 15.2 (14.8-15.5) |
| South | 2008 | 14.6 (14.3-14.9) |
| South | 2009 | 14.4 (14.1-14.7) |
| South | 2010 | 14.2 (13.9-14.5) |
| South | 2011 | 9.2 (8.9-9.4) |
| South | 2012 | 9.4 (9.2-9.7) |
| South | 2013 | 25.2 (24.8-25.6) |
| South | 2014 | 26.4 (26-26.8) |
| South | 2015 | 27.3 (26.9-27.6) |
| South | 2016 | 29.8 (29.4-30.2) |
| South | 2017 | 32 (31.6-32.4) |
| South | 2018 | 33.8 (33.4-34.2) |
| South | 2019 | 37.2 (36.7-37.6) |
| South | 2020 | 47.8 (47.3-48.3) |
| South | **Total** | 21 (20.9-21.1) |
| West | 1999 | 8.3 (7.9-8.6) |
| West | 2000 | 8.9 (8.5-9.2) |
| West | 2001 | 8.6 (8.2-8.9) |
| West | 2002 | 9.2 (8.9-9.6) |
| West | 2003 | 9.8 (9.5-10.2) |
| West | 2004 | 9.8 (9.4-10.1) |
| West | 2005 | 10.3 (9.9-10.6) |
| West | 2006 | 14.8 (14.4-15.3) |
| West | 2007 | 14.7 (14.3-15.1) |
| West | 2008 | 14.6 (14.2-15) |
| West | 2009 | 14 (13.6-14.3) |
| West | 2010 | 14 (13.6-14.4) |
| West | 2011 | 10.8 (10.5-11.1) |
| West | 2012 | 10.8 (10.5-11.1) |
| West | 2013 | 28.5 (28-29.1) |
| West | 2014 | 28.8 (28.3-29.3) |
| West | 2015 | 30.6 (30-31.1) |
| West | 2016 | 31.7 (31.2-32.3) |
| West | 2017 | 34.2 (33.6-34.7) |
| West | 2018 | 34.7 (34.2-35.3) |
| West | 2019 | 36.1 (35.5-36.6) |
| West | 2020 | 43.7 (43.1-44.3) |
| West | **Total** | 21.2 (21.1-21.3) |
| **Total** | **Total** | 19.1 (19.1-19.1) |

**Supplemental Table 8** Hypertension-related ESRD Age-Adjusted Mortality Rates per 100,000, Stratified by Urban-Rural Classification in Adults in the United States, 1999 to 2020.

| **Age-Adjusted Rate (95% CI)** | | |
| --- | --- | --- |
| **Year** | **Metropolitan** | **Nonmetropolitan** |
| 1999 | 9.6 (9.5-9.8) | 9.8 (9.4-10.2) |
| 2000 | 8.8 (8.6-8.9) | 7.1 (6.8-7.4) |
| 2001 | 8.7 (8.5-8.8) | 6.9 (6.6-7.3) |
| 2002 | 9.1 (8.9-9.2) | 7.3 (7-7.7) |
| 2003 | 9.6 (9.4-9.8) | 7.7 (7.3-8) |
| 2004 | 9.6 (9.4-9.8) | 8 (7.7-8.4) |
| 2005 | 9.9 (9.8-10.1) | 8 (7.6-8.3) |
| 2006 | 14.2 (14-14.4) | 12.6 (12.2-13) |
| 2007 | 13.6 (13.4-13.8) | 13.1 (12.7-13.5) |
| 2008 | 13.4 (13.2-13.6) | 12.1 (11.7-12.5) |
| 2009 | 13 (12.8-13.2) | 12.6 (12.2-13) |
| 2010 | 12.8 (12.7-13) | 12.5 (12.1-12.9) |
| 2011 | 8.9 (8.7-9) | 9 (8.6-9.3) |
| 2012 | 9 (8.9-9.2) | 8.7 (8.4-9) |
| 2013 | 24.4 (24.2-24.7) | 25.7 (25.1-26.3) |
| 2014 | 25.3 (25.1-25.6) | 25.7 (25.1-26.2) |
| 2015 | 26.5 (26.2-26.8) | 27.6 (27-28.2) |
| 2016 | 28 (27.8-28.3) | 29.9 (29.3-30.5) |
| 2017 | 30 (29.7-30.3) | 32.4 (31.8-33.1) |
| 2018 | 31.3 (31-31.5) | 34.6 (34-35.2) |
| 2019 | 33.3 (33.1-33.6) | 37.8 (37.1-38.5) |
| 2020 | 42.8 (42.5-43.1) | 48.5 (47.7-49.2) |
| Total | 19.1 (19.1-19.2) | 19 (18.8-19.1) |

**Supplemental Figure 1** Age-Adjusted Mortality Rates per 100,000 for Cardiovascular Deaths Related to Hypertension-related ESRD in Which the Underlying Cause of Death Was Restricted to CVD in Adults in the United States, 1999 to 2020

APC = annual percent change; CVD = cardiovascular disease; HRD= hypertensive renal disease.
